# Risky Music Listening, Permanent Tinnitus and Depression, Anxiety, Thoughts about Suicide and Adverse General Health

**DOI:** 10.1371/journal.pone.0098912

**Published:** 2014-06-04

**Authors:** Ineke Vogel, Petra M. van de Looij-Jansen, Cathelijne L. Mieloo, Alex Burdorf, Frouwkje de Waart

**Affiliations:** 1 Dept of Public Health, Erasmus MC University Medical Center, Rotterdam, the Netherlands; 2 Dept of Youth Policy, Municipal Public Health Service for Rotterdam Area, Rotterdam, the Netherlands; UNLV, United States of America

## Abstract

**Objective:**

To estimate the extent to which exposure to music through earphones or headphones with MP3 players or at discotheques and pop/rock concerts exceeded current occupational safety standards for noise exposure, to examine the extent to which temporary and permanent hearing-related symptoms were reported, and to examine whether the experience of permanent symptoms was associated with adverse perceived general and mental health, symptoms of depression, and thoughts about suicide.

**Methods:**

A total of 943 students in Dutch inner-city senior-secondary vocational schools completed questionnaires about their sociodemographics, music listening behaviors and health. Multiple logistic regression analyses were used to examine associations.

**Results:**

About 60% exceeded safety standards for occupational noise exposure; about one third as a result of listening to MP3 players. About 10% of the participants experienced permanent hearing-related symptoms. Temporary hearing symptoms that occurred after using an MP3 player or going to a discotheque or pop/rock concert were associated with exposure to high-volume music. However, compared to participants not experiencing permanent hearing-related symptoms, those experiencing permanent symptoms were less often exposed to high volume music. Furthermore, they reported at least two times more often symptoms of depression, thoughts about suicide and adverse self-assessed general and mental health.

**Conclusions:**

Risky music-listening behaviors continue up to at least the age of 25 years. Permanent hearing-related symptoms are associated with people’s health and wellbeing. Participants experiencing such symptoms appeared to have changed their behavior to be less risky. In order to induce behavior change *before* permanent and irreversible hearing-related symptoms occur, preventive measurements concerning hearing health are needed.

## Introduction

According to the World Health Organization, adult onset hearing loss (HL) is the second leading cause of ‘years lived with disability’ (YLD) after depression at global level, accounting for 4.6% of total global YLDs [1]. Exposure to excessive noise is a major cause of hearing disorder worldwide; 16% of the disabling HL in adults is attributed to occupational noise [2].

Outside the workplace millions of adolescents and young adults are potentially at risk of permanent hearing symptoms through listening to their favorite music. The risks of excessive music-listening are most likely to result in hearing-related symptoms such as tinnitus – a perceived sound in the ears with no external source of sound being present [3]–[5]. Prevalence of permanent tinnitus increases with age; previous research indicates that only 1% of those under 45 years experience permanent tinnitus, while about 12% of those aged 60–69 years develop it [6]. However, it has been reported that increasing numbers of adolescents and young adults now experience permanent symptoms indicative of poor hearing due largely to listening to music at high volumes [2], [7], [8].

It has been estimated that about 20% of Dutch adolescents aged 12–16 years are potentially at risk of developing hearing-related symptoms after 5 years because of listening to potentially hazardous music levels. Between 30.0% and 61.2% of them reported temporary hearing-related symptoms such as tinnitus after exposure to music from MP3 players and at discotheques [9]. If such exposure continues after the age of 16 years, temporary hearing-related symptoms will become more severe and even permanent.

Symptoms of poor hearing may lead to difficulties in future life. Because HL may influence communication and behavioral skills, it can adversely affect education and quality of life. Also, it is a growing social problem as more and more young people are limited in their choice of or even rejected from jobs because of preventable HL. Furthermore, it may lead to reduced psychological and social function, such as increased feelings of isolation, depression, loneliness, anger, fear, frustration, stress and disappointment [2], [8], [10].

Among 12–16 year olds, mainly adolescents attending pre-vocational education report relatively high levels of music exposure [11], [12]. Therefore, to investigate the prevalence of permanent hearing-related symptoms and its potential negative consequences among young people, we conducted a study among 16–25 year old students from lower educational levels. This group of students is assumed to be most and long enough exposed to potentially hazardous music levels, and thus the group most likely to experience permanent hearing-related symptoms such as tinnitus, muffled sounds, distortion or hyperacusis. We (1) estimated the extent to which these students’ exposure to music through earphones or headphones with MP3 players or at discotheques and pop/rock concerts exceeded current occupational safety standards for noise exposure, (2) examined the extent to which these students reported temporary and permanent hearing-related symptoms, and (3) examined whether the experience of permanent symptoms was associated with adverse perceived general and mental health, symptoms of depression, and thoughts about suicide.

## Methods

### Ethics Statement

According to the Dutch Act on public health, the Municipal Executive has to acquire, based on epidemiological analysis, insight into the health situation of the population and has to take care of systematic monitoring and highlighting of developments in the health status of young people [13]. All data were gathered through questionnaires by the local Municipal Public Health Service within this government-approved research of youth healthcare. Administration of the questionnaires at schools was carried out by specially trained researchers and public health promoters of the Municipal Public Health Service and/or a teacher. Students received written and verbal information about the study and were free to refuse to participation. The research was conducted in accordance with the requirements of the Dutch act on protection of personal data. Only anonymous data were used and the questionnaires were completed on a voluntary basis by students. Informed consent from parents is not required for people aged 16 and over [14]. Observational research with data does not fall within the ambit of the Dutch Act on research involving human subjects and does not require the approval of an ethics review board [15]. This study conformed to the principles embodied in the Declaration of Helsinki [16].

### Study Population and Procedure

A total of 1228 students (aged about 16–25 years) of 2 Dutch inner-city senior-secondary vocational schools were invited to complete questionnaires (Table S1) about their sociodemographics, music listening behaviors and health under supervision at school. All students that were present at the time of assessment completed the questionnaire. However, 272 students did not complete the questionnaire because of illness (27), visiting a doctor or nurse or stay away without leave (46), or because they were absent for unknown reasons (199); resulting in a total of 956 questionnaires, for a response of 77.9%. We excluded 13 more questionnaires because of an age outside the range of 16–25 years; thus, 943 questionnaires were used in the analyses. Participants’ ages ranged from 16 to 25 years (mean = 18; SD = 2). Sixty three percent were female, 66.3% were of non-Dutch ethnicity and 79.0% were living with their parent(s). Table 1 gives an overview of the socio-demographic characteristics of the study population. This table is adjusted from a previous study [17].

**Table 1:** Socio-demographic characteristics of study population (N = 943).

	Frequency in study population (unless otherwise specified)	
Mean age (years)	18 (sd 2; range 16–25)	
Gender		
Female	593	(62.9%)
Ethnicity		
Non-Dutch	625	(66.3%)
Home situation		
Living with parent(s)	745	(79.0%)

Characteristics adjusted from Vogel et al [17].

### Measures

Table S1 lists the survey items.

#### Sociodemographic characteristics

Sociodemographic characteristics included sex, age, ethnicity (Dutch; non-Dutch) and home situation (living with parent(s); not living with parent(s)). Ethnicity was determined on the basis of mother’s and father’s country/countries of birth according to definitions of Statistics Netherlands [18].

#### Risky music listening

Estimation of risky music listening behaviors – MP3 player listening and discotheque and pop/rock concert attendance – previously has been described in detail [9], [17]. Average weekly exposure times to MP3 players and stereos were estimated by multiplying days per week and hours per day. Average exposure time per month during discotheque visits was estimated by multiplying the number of discotheque visits per month by average time spent per visit. Average exposure time per year during pop/rock concerts was estimated by multiplying numbers of visits per year by 2.5 hours.

Since we did not measure music volume levels, to be able to estimate the exposure to potentially hazardous music levels, we had to estimate the music-volume levels per music source. Portnuff and Fligor have evaluated the output levels of several most popular players [19]. We used their grand average output levels across all evaluated players, which are similar to estimations made by a Dutch evaluation of the output levels of MP3 players and earphones [20], to convert volume-control levels of MP3 players and stereos into decibel levels. For discotheques the average decibel level was assumed to be 100 dBA, for pop/rock concerts 105 dBA [21].

Within current European occupational safety standards [22], music volume levels equal to or exceeding the equivalent of 80 decibels (dB/dBA) for 40 hours per week are assumed to be potentially damaging. However, in the report of the Scientific Committee on Emerging and Newly Identified Health Risks it is assumed that listening for 1 hour a day to a sound level of over 89 dBA is potentially damaging [2]. By applying the principle that a doubling in level (+3 dB) can be offset by halving the permissible exposure duration [23], [24], it can be calculated that listening 7 hours per week to a music level of 89 dB is equal to listening for 56 hours per week, or 8 hours per day, to a music level of 80 dBA. Therefore, we choose to use a loosened 56-hours criterion instead of the more stringent safety standard of 40 hours; i.e. 16 hours are added for the weekend days, because music listening is not restricted to working days.

To be able to estimate a weekly music dose (D) on the basis of reported exposure times and levels, we first calculated permissible exposure limits (PELs) for the estimated dBA levels of each participant per music source (MP3-player use and discotheque and pop/rock concert attendance) using the equation: PEL_(week)_ = 56/2^(L-80)/3;^ L stands for the estimated dBA level [24]. Secondly, each adolescent’s actual exposure time per music source was divided by the PEL to compute his or her estimated weekly music dose per music source [24]. Thirdly, the estimated doses per music source were summed to calculate an estimated total weekly music dose for all music sources combined [24]. A dose that was missing for a certain source was assumed to be zero.

To evaluate potential risk behavior, we first dichotomized responses on the basis of the loosened safety standard into students who were estimated to not be exposed to potentially hazardous music levels (D<1; exposed to equivalent levels of <80 dBA during 8 hours per day) and those who were estimated to be exposed to potentially hazardous music levels (D≥1; exposed to equivalent levels of ≥80 dBA during 8 hours per day) [24]. As an additional quantification of the severity of the potential risk, we applied three categories that estimated whether students were exposed (by source and by all sources combined) to a potential hazardous level for 8 hrs per day equivalent to: 80–85 dBA (low risk); 85–90 dBA (moderate risk); ≥90 dBA (high risk).

#### Health indicators

Hearing symptoms included symptoms such as tinnitus, muffled sounds, distortion, hyperacusis or (temporary) hearing loss. Temporary hearing-related symptoms were categorized as ‘I experienced hearing-related symptoms at least once in the past month after listening to an MP3 player’ (yes; no) and ‘I experienced hearing-related symptoms at least once in the past year after visiting discotheques and/or pop/rock concerts’ (yes; no). Permanent hearing-related symptoms were categorized as ‘I am constantly experiencing hearing symptoms’ (yes; no).

‘General health’ was categorized as: ‘My health in general is good or very good’ (yes/no). ‘Mental health’ was measured with the five-item Mental Health Inventory (MHI-5) [25]. The MHI-5 measures general mental health and can be used to screen for depressive symptoms and feelings of anxiety [26]. Each item asks the respondent about a particular feeling during the past month. The duration is reported on a six-point scale ranging from ‘all of the time’ to ‘none of the time’. Therefore, the score for each individual ranges between 5 and 30. This score is transformed into a variable ranging from 0–100 using a standard linear transformation, where a score of 100 represents optimal health. We used a cut-off score of <60 to define moderate-to-poor mental health. This cut-off is widely used and provides the best sensitivity and specificity [25], [27].

‘Symptoms of depression’ were measured with the Center for Epidemiologic Studies Depression Scale (CES-D) [28]. The CES-D was designed to measure current level of depressive symptomatology. It consists of 20 items that ask the respondent to indicate the frequency with which he/she experienced a symptom during the past week. One symptom, for example, is: ‘During the past week I did not feel like eating; my appetite was poor’. The frequency is reported on a 4-point response scale ranging from 0 (rarely/none of the time; less than 1 day) to 3 (most/all of the time; 5 to 7 days). Therefore, the summative score ranges between 0 and 60. Scores of ≥16 are regarded as clinically significant levels of depressive symptoms in the general population [28]. ‘Thoughts about suicide’ were categorized as: ‘I often or very often seriously thought to end my life during the past 12 months’ (yes/no).

### Statistical Analysis

Cross-sectional statistical analyses were performed using the SPSS program (version 19; SPSS Inc, Chicago, IL). Frequency tables were used to explore music listening, risky music listening and health of the total study population (N = 943), and females (n = 593) and males (n = 350); frequency differences were examined through chi-square statistics. Multivariate odds ratios (ORs) and their 95% confidence intervals (CIs) were calculated with multiple logistic regression analyses to explore the association between the estimated exposure to high-volume levels and both reported temporary and permanent hearing-related symptoms. Frequency tables and multiple logistic regression analyses were used to explore the associations between permanent hearing-related symptoms and the other health indicators (adverse perceived general and mental health, symptoms of depression, and thoughts about suicide). Any *p* values of <0.05 were considered to be statistically significant.

## Results

### Risky Music Listening

We estimated that 19.2% of participants were exposed to high-risk sound levels; they exceeded a sound level equivalent to 90 dBA for 56 hours per week for all sources of music combined; with regard to MP3 players alone, that figure was 15.4%, and for discotheques and pop/rock concerts 3.1% (Table 2).

**Table 2:** Music exposure of study population^a^ (*N* = 943).

	Total	Males	Females	*P* b
	*N* = 943	*n* = 350	*n* = 593	
**Music exposure**				
Listened to music on MP3 players (in past month)	88.0	83.6	90.7	***
Frequency of use				
7 d/wk (in past month)	35.4	34.6	35.9	
>3 h/d	15.4	17.9	14.0	
Used volume setting of more than three fourths	44.6	40.3	47.0	*
Visited discotheques (last year)	61.2	64.6	59.2	
Visited pop/rock concerts (last year)	39.8	43.2	37.9	
**Risky music exposure** c				
Risky exposure from combined sources (≥80 dBA)	61.5	64.0	59.9	
Low risk (80–85 dBA)	25.8	25.6	25.6	
Moderate risk (85–90 dBA)	16.5	18.7	15.3	
High risk (≥90 dBA)	19.2	19.6	18.9	
Risky exposure from MP3 players (≥80 dBA)	30.4	28.8	31.4	
Low risk (80–85 dBA)	11.6	13.8	10.3	
Moderate risk (85–90 dBA)	3.5	2.3	4.2	
High risk (≥90 dBA)	15.4	12.7	16.9	
Risky exposure from discotheques and pop/rock concerts (≥80 dBA)	48.1	54.8	44.2	***
Low risk (80–85 dBA)	30.1	30.0	30.0	
Moderate risk (85–90 dBA)	15.0	19.6	12.3	
High risk (≥90 dBA)	3.1	5.2	1.9	

a Values reported are percentages.

b Females compared to males.

*P<0.05.

***P<0.001.

c Average exposure equivalent to sound levels of 80 dBA during ≥56 hours per week.

### Health Indicators

After listening to music on MP3 players 14.5% experienced temporary hearing-related symptoms; after visiting discotheques and/or pop/rock concerts 44.2%. Almost 10% of participants reported constant hearing-related symptoms; about one third of them consulted a physician for their hearing problems. Regarding the other four health indicators, (adverse perceived general health, adverse mental health, depressive symptoms and thoughts about suicide) frequencies for experiencing adverse outcomes ranged between 17.8% (thoughts about suicide) and 34.5% (mental unhealthy). For all indicators, in comparison with males, females reported more often adverse outcomes (Table 3).

**Table 3:** Health indicators of study population^a^ (N = 943).

Health indicators	Total	Males	Females	*P* b
	*N* = 943	*n* = 350	*n* = 593	
Temporary hearing-related symptoms^c^				
At least once in the prior month after listening to music on an MP3 player	14.5	14.1	14.7	
In the past year after going to a discotheque or pop/rock concert	44.2	46.7	43.0	
Permanent hearing-related symptoms^c^	9.2	8.1	9.9	
Consulted a physician for hearing	2.9	2.0	3.4	
Adverse perceived general health	30.9	22.2	36.1	***
Adverse mental health	34.5	25.9	39.5	***
Depressive symptoms	32.7	23.3	38.3	***
Thoughts about suicide (past year)	17.8	12.4	21.1	***

a Values reported are percentages.

b Females compared to males *P<0.05, ***P<0.001

c Hearing-related symptoms included symptoms such as tinnitus, muffled sounds, distortion, hyperacusis or (temporary) hearing loss.

### Risky Music Listening and Hearing-related Symptoms

Temporary hearing-related symptoms – such as tinnitus, muffled sounds, distortion, hyperacusis or hearing loss – that occurred after using an MP3 player or going to a discotheque or pop/rock concert were associated with exposure to high-volume music (Table 4). Students that reported listening at high-risk levels experienced almost two times more often temporary hearing-related symptoms than students listening at sounds levels that were not risky. For discotheques and pop/rock concerts this was more than 15 times more often.

**Table 4:** Prevalence (%) of self-reported hearing-related symptoms and the association with estimated exposure to potentially hazardous music levels.

	Temporary hearing-related symptoms (N = 856)^a^				Permanent hearing-related symptoms (N = 943)	
	MP3 players		Discotheques and pop/rock concerts			
	Frequency of hearing symptoms such as tinnitus ‘at least once in the past month’		Frequency of hearing symptoms such as tinnitus ‘at least once in the past year’			
	%	OR^b^ (95% CI)	%	OR^b^ (95% CI)	%	OR^b^ (95% CI)
Total study population	16.0^a^		48.7^a^		9.2	
**Music exposure**						
Not at risk – reference (<80 dBA^c^)	14.7	1.00	24.6	1.00	10.7	1.00
Low risk (80–85 dBA^c^)	17.0	1.25 (0.69–2.25)	73.2	8.84 (6.18–12.63)***	9.9	0.86 (0.49–1.49)
Moderate risk (85–90 dBA^c^)	9.7	0.62 (0.18–2.09)	74.2	8.69 (5.49–13.76)***	10.3	0.93 (0.50–1.75)
High risk (≥90 dBA^c^)	22.3	1.70 (1.07–2.70)*	82.1	15.30 (5.62–41.65)***	4.4	0.39 (0.18–0.86)*

OR = Odds Ratio; CI = Confidence Interval; dBA = decibels.

a Participants with reported permanent hearing symptoms excluded (N = 87).

b Adjusted for age and gender.

c Equivalent sound level in dBA for 56 hrs per week.

*P<0.05.

***P<0.001.

Compared to students experiencing such symptoms, students not experiencing permanent hearing-related symptoms listened more than 2.5 times more often to sound levels equivalent to ≥90 dBA for 56 hours per week (high-risk sound levels) (Table 4).

### Permanent Hearing-related Symptoms and other Health Indicators

All other health indicators – adverse perceived general health, adverse mental health, depressive symptoms and thoughts about suicide – were significantly related to permanent hearing-related symptoms (corrected for sociodemographics). Compared to participants not experiencing permanent hearing-related symptoms, those experiencing such symptoms reported at least two times more often symptoms of depression, thoughts about suicide and adverse self-assessed general and mental health (Table 5).

**Table 5:** Prevalence (%) of self-reported other adverse health indicators and the association with experiencing permanent hearing-related symptoms (*N* = 943).

	Permanent hearing-related symptoms	
	%	OR^a^ (95% CI)
Total study population	9.2	
**Health indicators**		
Adverse perceived general health	47.1	2.03 (1.26–3.25)**
Adverse mental health	55.2	2.68 (1.66–4.33)***
Depressive symptoms	56.3	2.58 (1.60–4.16)***
Thoughts about suicide (past year)	31.0	1.99 (1.19–3.34)**

OR = odds ratio; CI = confidence interval.

a Adjusted for age, sex, ethnicity and home situation.

**P<0.01.

***P<0.001.

## Discussion

An alarming percentage of almost 10% of this relatively young age group reported permanent hearing-related symptoms. Furthermore, experiencing such symptoms was related to adverse self-assessed general and mental health, and to having symptoms of depression and thoughts about suicide.

Our study is one of the first to provide a preliminary insight into the association between exposure of 15–25-year-old inner-city youth attending lower education to potentially hazardous music and temporary and permanent hearing symptoms, and into the association between permanent hearing-related symptoms and adverse health consequences. The results show that risky music-listening behaviors continue up to at least the age of 25 years among students attending lower educational levels. We estimated that by listening to high-volume music during leisure time, about 20% listened on average to sound levels that may cause permanent hearing-related symptoms such as tinnitus or even permanent hearing loss that is noticeable to individual people themselves after 5 years of such exposure [29].

As found previously [9], [30], this study showed that participants exposed to potentially hazardous sound levels experienced temporary hearing-related symptoms more often than those not exposed to such sound levels. Most people tend to underestimate the threat of music-induced hearing loss, probably because of the gradual development of hearing loss and because most people with mild high-frequency hearing loss are unaware of their impairment [31]. Repeated experiences of temporary hearing-related symptoms might be an indication that an individual’s hearing is susceptible to noise-related damage and should be taken as warning signs for people to reduce their exposure to very high noise levels [31], [32]. However, previous research has shown that young people attending lower educational levels who had experienced tinnitus did not consider this as a warning that their hearing was susceptible to damage from loud music and did not intend to change their behavior voluntarily [11], [12], [33].

Remarkably, most of the alarming percentage of almost 10% of this relatively young age group that reported permanent and thus irreversible hearing-related symptoms appeared to have changed their behavior: the more often permanent hearing-related symptoms were reported, the lower the reported average sound levels. Probably these people adjusted their behavior in order to prevent the symptoms from worsening and protect their hearing from further deterioration. Furthermore, experiencing permanent symptoms was related to adverse self-assessed general and mental health, and to having symptoms of depression and thoughts about suicide. These results show that permanent hearing-related symptoms occur considerably among certain groups of youth and are associated with young people’s health and wellbeing comparable to what has been found among adults [6].

There is previous evidence that hearing problems are associated with depression and other serious mental health problems. An Australian study found that compared with population norms, hearing disability at all levels was associated with poorer mental health [34]. A meta-analysis reported that hearing impairment (HI) is among the most common chronic conditions associated with depression [35]. A systematic review of the literature demonstrated that HI children and adolescents were more prone to outcomes with lower quality of life and more mental health problems than normally hearing (NH) children and adolescents. For example, HI individuals have more difficulties with making friends and are more socially isolated. It also consistently demonstrated that HI children en adolescents were more prone to developing depression, aggression, oppositional defiant disorder, conduct disorder, and psychopathy than their normally hearing peers [36]. A recent study [37] found - after accounting for health conditions and other factors - a strong association between HI and depression among US adults of all ages. The prevalence of depression increased as HI became worse, except among participants who, by self-report, were deaf and least likely to report depression [37].

On the other hand, previous studies have reported a reduction in depressive symptoms of people using hearing aids [38]–[40]. The finding that treated hearing loss improves mental health might be an indication that at least a part of mental health problems could be avoided by preventing hearing impairment. In combination with our results, this implies that hearing symptoms in youth need to be addressed and appropriately managed [41] and, preferably, be prevented to avoid such adverse consequences. In order to induce behavior change *before* permanent and irreversible hearing-related symptoms occur, we recommend that health authorities, those involved in presenting music venues, schools, parents, and young people should be informed about the potential risks of high-volume music via public health campaigns and the mass media, and should be alerted to the need for preventive hearing healthcare by health professionals such as practitioners of pediatrics, family medicine, audiology and youth health care. Real examples of peer-group people who had lost hearing through listening to loud music should be provided [33]. Previous research identified the following opportunities for protective measures concerning potentially hazardous music in music venues [21]: music venues could inform their visitors for the potential risk for hearing loss and inform and warn visitors about the potential dangers of exposure to high-volume music by clearly stating this on their advertisements, at the venue entrance, and on the tickets for admission. Furthermore, they could make available ‘ear rest rooms’ (areas with low-volume music), as well as hearing protection devices.

This study has several limitations. One was the use of a convenience sample of students attending senior-secondary vocational education. However, most of the characteristics of the study group reflected those of this subgroup of Dutch inner-city adolescents and emerging adults. The group of non-Dutch ethnicity (66.3%) consists of the following ethnicities: Surinam (15,3%) Antillean (9,0%) Moroccan (12%) Turkish (15,0%) other (15%). These percentages reflect those of the population of the Rotterdam inner-city senior-secondary vocational schools. Although the proportion of females attending inner-city senior-secondary vocational education is somewhat greater than the proportion of males, the proportion of females was relatively greater in our sample [18]. Also, we have no information about the music-listening behaviors of nonparticipants in the study. With regard to selective nonresponse, our nonparticipation was 22.1%; this may have affected the results. The data used in our study were cross-sectional and self-reported, which implies that no causal relationships can be inferred [42].

We did not report on the relation between drug use and both auditory symptoms and the other adverse health indicators. Drug use might increase the risk of noise induced hearing loss and/or other adverse health indicators [43], [44]. However, after checking, we found no significant association between permanent hearing symptoms and both cannabis use and hard drug use in the past four weeks. We reanalyzed the data by additionally correcting the multiple logistic regression analyses of Table 5 for cannabis and hard drug use in the past four weeks. This resulted in only marginally lower odds ratios (results not shown).

We may have underestimated the exposure levels for several reasons. First, because we did not take into account other potentially hazardous sounds to which the participants in our study may have been exposed, which increase the risk of hearing loss. In a previous study we found that adolescents were also exposed to other potentially hazardous sounds, such as music through loudspeakers (88.0%) and through their own activities as musicians (21.5%), as well as the noise to which they were exposed when riding mopeds or scooters (27.1%) or when using firecrackers (60.8%) [9]. Future studies should also take account of such exposures. Furthermore, we did not ask participants whether they used earbud-style earphones or supra-aural earphones. The output level should be corrected by adding 5.5 decibels when using earbud-style earphones [2], [45] and previously has been found that 93.2% of adolescents used earbud-style earphones [46]. Finally, in the absence of safety standards for leisure-time sound exposure, we relied on loosened occupational safety standards. However, there is no scientific evidence that social noise produces different NIHL levels compared to occupational noise [2], [47].

In conclusion, as hearing problems tend to isolate people from friends, family and others because of a decreased ability to communicate, the impact of HI may be profound as it can impose a heavy social and economic burden on individuals, families, communities, and countries [37]. Our results show the need for structural action by both attention for future research regarding the consequences of music-listening behaviors for hearing, as well as for the development and implementation of strategies for prevention and intervention. This would also contribute to the development of safety standards for leisure-time noise exposure. If these findings are confirmed, such standards are essential to avoid the possibility that entire generations could be suffering severe hearing problems due to excessive high-volume music exposure in their childhood, adolescence and emerging adulthood.

## Supporting Information

Table S1 Survey items.
(DOC)
