# Supplementary material for: Risky Music Listening, Permanent Tinnitus and Depression, Anxiety, Thoughts about Suicide and Adverse General Health
Source: PLoS One. 2014 Jun 4;9(6):e98912. doi: 10.1371/journal.pone.0098912 (PMC4045887; doi:10.1371/journal.pone.0098912)
Supplement: Table S1 — Survey items. (DOC) [file pone.0098912.s001.doc]

| **Table S1.** Survey items | |
| --- | --- |
|  |  |
| **1** | **Socio-demographic characteristics** |
|  | - Are you a boy or a girl? |
|  | - What is your age in years? |
|  | - What is your country of birth? |
|  | - What is your father’s country of birth? |
|  | - What is your mother’s country of birth? |
|  | - With whom do you share a household? participant shared a household with at least one of his or her own parents (yes; no) |
| **2** | **Music listening behavior** |
|  | ***MP3 player*** |
|  | - Do you ever listen to music through earphones on a portable music player (MP3 player)? |
|  | - On average over the last month, on how many days per week did you listen to music on an MP3 player? |
|  | - How long do you normally use your MP3 player per day? |
|  | - At what volume-control level do you normally listen? |
|  | *Discotheque* |
|  | - Have you been to a discotheque in the last year? |
|  | - On average over the last year, how many times per month did you go to a discotheque? |
|  | *Pop concert* |
|  | - During the past year, how often did you go to a pop concert? |
| **3** | **Health indicators** |
|  | - On average over the last month, how often did you experience hearing symptoms such as tinnitus, muffled sounds, distortion, hyperacusis or temporary hearing loss after listening to music on an MP3 player? |
|  | - After going to the discotheque, how often did you experience hearing symptoms such as tinnitus, muffled sounds, distortion, hyperacusis or temporary hearing loss? |
|  | - After going to a pop concert, how often did you experience hearing symptoms such as tinnitus, muffled sounds, distortion, hyperacusis or temporary hearing loss? |
|  | - Do you constantly experience hearing symptoms such as a ringing sound in your ears, hearing loss, hypersensitivity to sounds or muffled sounds? |
|  | - How do you rate your health in general? |
|  | - During the past 12 months how often did you seriously think to end your life? |
